# iPSC-MSCs and islet allograft tolerance

**DOI:** 10.18632/oncotarget.4050

**Published:** 2015-05-10

**Authors:** Tong-Song Wang, Pan-Pan Cheng, Zhong-Quan Qi

**Affiliations:** Qingdao Municipal Centers for Disease Control and Prevention, Qingdao, Shandong, PR China

The immunosuppressive effect and low immunogenicity of mesenchymal stem cells (MSCs) make them ideal candidates for immunosuppressive strategies. Adult MSCs have been applied widely in organ transplantation. However, their limited accessibility limits their application in the clinic. Furthermore, successive passages slow the proliferation rate, reduce the multipotency and lower the immunosuppressive activity of adult MSCs in culture.

Induced pluripotent stem cells-derived MSCs (iPSC-MSCs) may provide an alternative source of MSCs. iPSC-MSCs have the same characteristics as adult MSCs and exhibit great immunomodulatory properties. For example, iPSC-MSCs displayed significant inhibition of NK cell proliferation and cytolytic function [1]. iPSC-MSCs modulated T-cell phenotypes towards Th2 suppression and induced T regulatory cell (Treg) expansion in the peripheral blood from allergic rhinitis patients [2]. And the similar therapeutic effects on Th2 cytokines were observed in a mouse model of ovalbumin-induced allergic inflammation [3].

Given the great immunomodulatory properties of the iPSC-MSCs, we speculated that iPSC-MSCs could be a promising strategy for preventing transplant rejection. Indeed, we found that iPSC-MSCs combined with low-dose rapamycin (Rapa, 0.1 mg/kg/d, i.p., from day 0 to day 9) significantly prolonged islet allograft survival in the diabetic mice, and even induced immune tolerance in 50% recipients, though iPSC-MSCs alone was inefficient in preventing rejection [4]. We also found that production of interleukin-2 and interferon-γ was reduced, which indicated iPSC-MSCs + Rapa treatment reduces Th1 inflammatory cytokines and may modulate T-cell phenotypes towards Th1 suppression. However, the iPSC-MSCs modulated T-cell phenotypes towards Th2 suppression in allergic rhinitis patients and animal models [2, 3]. These results indicate that iPSC-MSCs maybe exerts different effects and activities in different pathological conditions. It allows the iPSC-MSCs to rectify the imbalance of the immune responses in different environments.

Previous studies have verified that adult MSCs alone, or combined with immunosuppressive drugs, induce allograft immune tolerance through Treg induction in vivo. Berman et al. first reported that infusions of donor or third-party MSCs prolonged islet function, which was associated with increased numbers of Tregs in peripheral blood [5]. We speculated that iPSC-MSCs exert the therapeutic effect possibly through the induction of Tregs, acting like adult MSCs. Since TGF-β is the perpetrator of immune suppression via regulatory T cells [6], we examined TGF-β expression in the islet grafts and sera of the recipient mice. The results showed that iPSC-MSCs + Rapa treatment increased sera TGF-β concentrations, and graft *TGF-β* mRNA levels, compared with Rapa treatment. Flow cytometry results also showed that the number of T regulatory cells was increased in spleen and lymph nodes in the iPSC-MSCs+ Rapa group compared with Rapa group. These results support our hypothesis.

In addition to 2,3-dioxygenase, prostaglandin-E2, nitric oxide, TGF-β, and hepatocyte growth factor, IL-10 has been reported to induce MSC-mediated immunosuppressive effects. IL-10 induced by CD11b(+) cells and IL-10-activated regulatory T cells played important roles in immune modulation of mesenchymal stem cells in rat islet allografts [7]. In our research, iPSC-MSCs alone or in the combination with Rapa increased IL-10 secretion in sera, and IL-10 mRNA levels in the graft, whereas Rapa alone did not. Further research is still required to determine whether IL-10 mediates the immunosuppressive effect of iPSC-MSCs + Rapa on T cells. In addition, transwell separation significantly weakened the immunosuppressive effects of iPSC-MSCs on the proliferation of Con A-treated splenic T cells, which revealed that the combined treatment exerts its immunosuppressive effects though cell-cell contact and the regulation of cytokines production.

Anti-aging and anti-cancer benefits of low-dose rapamycin were recently discussed [8]. The synergistic immunomodulatory effects of iPSC-MSCs and lowdose Rapa in islet transplantation suggest a promising strategy for inducing immune tolerance, not only in allogeneic islets transplantation, but also in other solid organ transplantation. There may be differences in immunosuppressive effects and mechanisms between iPSC-MSCs and MSCs from other sources. And the origin of the cell sources and the long-term effects of iPSC-MSCs also need to be investigated.
